# Diné traditional medicine use and wellbeing among navajo adults during the COVID-19 pandemic: A cross-sectional study

**DOI:** 10.1371/journal.pone.0337427

**Published:** 2025-12-05

**Authors:** Rachelle L. Begay, Heidi E. Brown, Priscilla R. Sanderson, Robin B. Harris

**Affiliations:** 1 Department of Epidemiology and Biostatistics, Mel and Enid Zuckerman College of Public Health, The University of Arizona, Tucson, Arizona, United States of America; 2 Department of Health Sciences, College of Health and Human Services, Northern Arizona University, Flagstaff, Arizona, United States of America; University of Arkansas for Medical Sciences, UNITED STATES OF AMERICA

## Abstract

**Objective:**

To assess perceptions of the impact of the COVID-19 pandemic on access, utilization, and perceived quality of Diné traditional medicine and healing (DTM) and psychosocial wellbeing among members of the Navajo Nation.

**Methods:**

From May to October 2021, a convenience sample of 153 self-identified Navajo participants were recruited to complete a cross-sectional survey. We developed the survey to gather sociodemographic participant information and explored the perceived impact of the COVID-19 pandemic on Navajo Nation members’ access, utilization, and quality of health care (including DTM practices), employment and finances, and psychosocial wellbeing. Data were analyses using descriptive statistics and Wilcoxon matched-pairs signed-rank test with comparisons made by DTM use.

**Results:**

We found that nearly half (n = 74, 48.7%) of the study sample reported use of Diné traditional medicine (DTM) or healing during the COVID-19 pandemic. There were no significant differences (P > 0.05) in history of a prior positive COVID-19 test between DTM users and non-users. DTM users indicated that the COVID-19 pandemic had minimal impact on the barriers or issues such as transportation, elder-/childcare or appointment availability they faced in receiving DTM care or healing (P > 0.05 on all pre- vs mid-pandemic scores) and scored higher on the COVID-19 Risk Factor Score than non-users (P = 0.01).

**Conclusion:**

Despite significant challenges imposed by the COVID-19 pandemic, Diné people were still able to access DTM. Diné traditional medicine and healing is a critical component of overall health and well-being for the Diné people.

## Introduction

On March 17, 2020, the first confirmed case of COVID-19 was reported on the Navajo Nation and subsequently linked to a church gathering attended by individuals from over half a dozen communities from across the Navajo Nation [1,2]. Within the first several months of local transmission in the United States (US), the Navajo Nation became a COVID-19 hotspot with per-capita infection rates far outpacing more densely populated areas in the US such as New York City and Los Angeles [1,3,4]. The Centers for Disease Control and Prevention (CDC) estimated that COVID-19 infection rates among American Indian and Alaska Natives AI/ANs was 3.5 (95% CI 1.2–10.1) times that of the non-Hispanic white population [5]. Additionally, case fatality rates (~4%) for the Nation were twice what was reported for neighboring Arizona and New Mexico, 2.0% and 2.1% respectively, during the same period [6].

The disproportionate burden of COVID-19 infection and mortality among the Navajo or Diné people, as well as other AI/AN communities in US, are legacies of centuries of broken treaties, structural racism, and settler colonialization [5,7,8]. The Snyder Act of 1921 and Indian Health Care Improvement Act of 1976 ordered treaty rights for quality healthcare for AI/AN nations in the US, treaties that remain unfulfilled to this day [8]. Decades of underfunding of public health infrastructure and the Indian Health Service have contributed to the higher prevalence of chronic disease including cancer, diabetes, hypertension, heart disease and kidney disease among the Navajo population – all of which are comorbid conditions linked to adverse COVID-19 clinical outcomes [9–11]. In fact, these additional burdens and the excessive transmission rates and their impact on our community health partners of the Navajo Healthy Stomach Project that brought this project into existence.

Likewise, settler colonial structures and forces designed for control of land and resources have created socioeconomic conditions and frayed infrastructure have made many households and communities on the Navajo Nation vulnerable to COVID-19 infection [8,12–14]. The Navajo Nation spans 27,000 miles and three states (Arizona, New Mexico, and Utah) in the Four Corners area of the US. It is home to approximately 400,000 people. However, in an area roughly the size of West Virginia, Diné citizens have access to only 13 grocery stores (driving an average of three hours for groceries) and just 13 health care facilities (12 on-reservation and one located off-reservation) [8,14]. Food insecurity and poor access to health care services have been linked to increased COVID-19 infection and mortality among other rural and minoritized populations in the US [15,16]. Moreover, along with high rates of unemployment and poverty, nearly 30% of the population does not have access to running water [14,17]. These conditions increased the difficulty for Navajo residents to adhere to COVID-19 mitigation advice including frequent handwashing.

Furthermore, until the passage of the American Indian Religious Freedom Act in 1978, AI/ANs were prohibited from and often punished for visiting sacred sites, performing religious dances, and conducting ceremonies and other traditional medicine practices [18]. However, despite these efforts, a 1998 study estimated that 39% of Navajo (Diné) patients at an Indian Health Services clinic reported being regular users of traditional medicine or native healers [19]. In Diné culture, traditional medicine (DTM) “sees a person not simply, as a body but as a whole being with body, mind, and spirit seen to be connected to other people, to families, to communities, and even to the planet and universe.” [20–23]. Indigenous health and well-being encompass more than simply the absence of disease or physical health but includes emotional, mental, and spiritual elements of life and extends beyond the individual to incorporate land, animals, family, and community [24]. On the Navajo Nation, the Diné Hataałii Association is a 501I(3) nonprofit organization established in the 1970s comprised of medicine men and women from across the Nation to protect, preserve and promote Diné traditional cultural wisdom, ceremony, and herbal healing knowledge [25]. The organization is comprised of spiritual leaders and cultural wisdom keepers that include but are not limited to Hataałii (medicine men and chanters), herbalists, diagnosticians, and singers.

At the height of the COVID-19 pandemic on Navajo Nation, leaders implemented nightly curfews and 57-hour weekend lockdowns from 8:00 pm on Friday through 5:00 am on Monday [26]. Residents were ordered to remain at home, with exceptions for certain essential workers, first responders, and health care personnel [27]. To address concerns raised by community health partners of our Navajo Healthy Stomach Project regarding the impact of COVID-19 mitigation strategies on Diné people’s access and utilization of allopathic medical care and DTM, we developed a 1-year multimethod study to explore the impact of the COVID-19 pandemic on Navajo community members’ livelihood including healthcare access (allopathic and DTM) and personal well-being, using online and in-person surveys and focus group discussions. Results from our primary analyses describing risk factors associated with COVID-19, utilization and access to allopathic health care during the pandemic and mechanisms of resilience among Navajo adults are described elsewhere [28].

In recognition that health is more than accessing allopathic medicine, this investigation sought to examine community members’ perceptions of the impact of the COVID-19 pandemic on access, utilization, and perceived quality of DTM on the Navajo Nation. Additionally, we characterize individuals who sought or used DTM during the COVID-19 pandemic, including their personal and psychosocial wellbeing. This work adds to our understanding of DTM use, especially during times of crisis.

## Methods

We recruited participants from 18 May to 12 October 2021 to complete a survey designed to explore community members’ perceptions of COVID-19 impacts on access, utilization, and quality of health care (allopathic and DTM) on the Navajo Nation. The surveys were conducted online or in-person.

### Study site

The Navajo Nation is the largest Indian reservation in the US. The area, in Northeast Arizona, Northwest New Mexico and Southeast Utah exceeds the size of ten other US states. The total population (2020 estimates) is 165,158 representing a population density of 6.0 per square mile and is considered rural using the Health Resources & Services Administration definitions of rural [29].

### Research approval process

Working with American Indian communities requires special Institutional Review Board (IRB) oversight and approval from the local communities [30]. The project team secured approval from local governing bodies and community stakeholders. We received approval resolutions from two Navajo Nation Agency Councils (Western and Fort Defiance), 16 chapters (local governing units) within those Agencies and from the Diné Hataałii Association (approved 01/24/2021). Study procedures were approved by the Navajo Nation Human Research Review Board (NNR-20.391 approved 03/15/2021) and the University of Arizona IRB (#2010166660 approved 11/18/2020). All participants signed a consent form (online for those accessing the survey online and on paper for those who were recruited in person). The draft of this manuscript was submitted to the NNHRRB (5/14/2024) and approval was received (1/17/2025). In addition, we established a Community Advisory Committee (CAC) of community members from various regions of Navajo Nation to provide feedback and oversight on study recruitment, methods, and dissemination.

### Study participants and data collection

Participants were recruited for the study using five convenience sampling methods: 1) a two-hour radio forum on Navajo Nation’s KTNN 600 AM radio station in May 2021, 2) recruitment flyers emailed to Navajo Nation chapters, 3) the research team’s website, and 4) social media, specifically Facebook and Instagram. Following the end of 57-hour stay-at-home lockdowns on Navajo Nation in July 2021, subjects were also recruited from in-person tabling at four outdoor chapter flea markets (July 2021 – September 2021). Participants were eligible to participate if they were at least 18 years of age and self-identified as Navajo.

Participants completed the COVID-19 Impact Survey which assessed a) COVID-19 exposure, symptoms, testing, and vaccination, b) impact on cancer care seeking, c) barriers to (allopathic) health care, including barriers introduced due to lockdown restrictions, d) barriers to Diné Traditional Medicine care, including barriers introduced due to lockdown restrictions and e) COVID-19 Well-being including psychosocial impact, f) resilience and coping, and g) employment and financial impact. Additionally, housing, living and community demographic questions were asked to understand the influence of these factors on care-seeking capacity. The questionnaire was reviewed by CAC members and the Diné Hataałii Association and edited according to feedback. A copy of the questionnaire is available in Supplemental material.

Surveys were administered through REDCap (Research Electronic Data Capture) electronic data capture tools hosted at the University of Arizona, which permitted secure data entry and met privacy requirements for research studies [31,32]. Additionally, a small team of University of Arizona and Northern Arizona University staff and students administered in-person surveys to participants recruited at the four outdoor chapter flea markets (July 2021 – September 2021). These surveys were then entered into REDCap by the research team.

Of the initial 190 participants recruited, 169 (89%) consented to participate in the survey. Most of the survey responses were entered by the research team from the paper forms (n = 127), with fewer respondents (n = 27) directly entering into the online forms. Only individuals that completed the entire survey (n = 153 or 81% of recruited participants) were included in this analysis. The survey allowed for participant non-response or refusal and these responses were coded as missing. Thus, missingness was observed in our final analytic sample, see Table 1.

**Table 1:** Characteristics of Navajo survey participants in the COVID-19 Impact Study (N = 153) by Diné Traditional Medicine (DTM) Use.

	Overall N = 153	DTM Use N = 74	No DTM Use N = 79	p value*
**Characteristics**	**n (%)**	**n (%)**	**n (%)**	
*Individual*				
**Age in years**, mean (SD)	44.65 (15.76)	41.28 (15.86)	48.44 (14.92)	0.02
**Age, categorized**				<0.01
Missing	51	20	31	
18 to <45	51 (50.0%)	35 (64.8%)	16 (33.3%)	
45 to <65	40 (39.2%)	13 (24.1%)	27 (56.2%)	
65 and older	11 (10.8%)	6 (11.1%)	5 (10.4%)	
**Gender**				0.60
Female	101 (66.4%)	47 (64.4%)	54 (68.4%)	
Male	51 (33.6%)	26 (35.6%)	25 (31.6%)	
**Education**				<0.01
Less than High School	24 (15.9%)	9 (12.2%)	15 (19.5%)	
High School Diploma or GED	32 (21.2%)	9 (12.2%)	23 (29.9%)	
More than High School Diploma/GED	95 (62.9%)	56 (75.7%)	39 (50.6%)	
**Employment Status**				<0.01
Not employed	45 (30.6%)	22 (29.7%)	23 (31.5%)	
Part Time or Full Time	72 (49.0%)	45 (60.8%)	27 (37.0%)	
Other	30 (20.4%)	7 (9.5%)	23 (31.5%)	
**Essential Worker Status**				0.95
Essential Health Care	6 (5.8%)	2 (3.9%)	4 (7.7%)	
Frontline Essential	30 (29.1%)	15 (29.4%)	15 (28.8%)	
Other Essential	12 (11.7%)	6 (11.8%)	6 (11.5%)	
> 1 category	23 (22.3%)	12 (23.5%)	11 (21.2%)	
Non-essential	32 (31.1%)	16 (31.4%)	16 (30.8%)	
*Household and Community*				
**Number of people in household**, mean (SD)	3.49 (1.78)	3.53 (1.76)	3.44 (1.82)	0.76
**Number of generations in household**				0.21
1 generation	45 (37.5%)	21 (33.3%)	24 (42.1%)	
2 generations	50 (41.7%)	25 (39.7%)	25 (43.9%)	
3 or more generations	25 (20.8%)	17 (27.0%)	8 (14.0%)	
**Housing Type**				0.03
Modern (HUD, non-HUD or NHA housing)	70 (46.1%)	41 (55.4%)	29 (37.2%)	
Modular house/ trailer	48 (31.6%)	18 (24.3%0	30 (38.5%)	
Traditional (Hogan or Rock)	10 (6.6%)	7 (9.5%)	3 (3.8%)	
Other	24 (15.8%)	8 (10.8%)	16 (20.5%)	
**Community Type**				0.15
Border Town	19 (12.6%)	10 (13.7%)	9 (11.5%)	
Rural (not many homes nearby, small town)	66 (43.7%)	38 (52.1%)	28 (35.9%)	
Town (in a smaller city or town)	50 (33.1%)	19 (26.0%)	31 (39.7%)	
Urban (in a large city)	16 (10.6%)	6 (8.2%)	10 (12.8%)	
**Distance to Grocery**				0.65
Less than 15 minutes	46 (30.5%)	21 (28.8%)	25 (32.1%)	
15–30 minutes	39 (25.8%)	19 (26.0%)	20 (25.6%)	
30 minutes to 1 hour	37 (24.5%)	16 (21.9%)	21 (26.9%)	
1 hour or more	29 (19.2%)	17 (23.3%)	12 (15.4%)	
*COVID-19 Experiences*				
**COVID-19 Testing**				0.40
Never Tested	42 (27.5%)	18 (24.3%)	24 (30.4%)	
All Negative Tests	64 (41.8%)	36 (48.6%)	28 (35.4%)	
Ever Positive	25 (16.3%)	13 (17.6%)	12 (15.2%)	
**COVID-19 Vaccination Status**				0.96
** **No, not vaccinated	23 (15.0%)	11 (14.9%)	12 (15.2%)	
** **Yes, at least one dose	130 (85.0%)	63 (85.1%)	67 (84.8%)	
**Job or income loss due to pandemic**	37 (24.2%)	23 (31.1%)	14 (17.9%)	0.09

* based on t-tests and chi-square tests; For the following variables, missingness was less than 5%: Gender (1 missing), Education (2 missing), Employment (6 missing), Housing Type (1 missing), Community Type (2 missing), Distance to Grocery (2 Missing), Job/Income Loss (1 missing). For the following variables, missingness was 14–33%: Essential worker (50 missing = 33%), # Generation in HH (33 missing = 22%, COVID-19 Testing (22 Missing = 14%).

### Measures

This analysis focused on COVID-19 Impact Survey questions regarding Diné traditional medicine (use and barriers) and COVID-19 well-being. The primary investigation of this study including analysis of the remaining survey items and focus group discussions are described in Brown et al., 2024 [28].

**Diné Traditional Medicine Use**. To assess Diné traditional medicine or healing use, participants were asked, “*Do you seek Dine traditional medicine or healing? (including medicine man, roadman, herbalist, diagnostician, or other traditional practitioner)”* and given the option of a yes or no response for each type of practitioner. A “yes” response indicated DTM use and a “no” response indicated no DTM use.

**Barriers to Diné Traditional Medicine and Healing.** To understand barriers or issues participants encountered in seeking DTM before compared to during the COVID-19 pandemic, participants were asked five potential barriers (transportation, money/finances, convenience, child/elder care, and practitioner knowledge) rated on a 5-point Likert scale from 0 = strongly disagree to 4 = strongly agree. Higher scores indicated participant experienced more of the barrier assessed. Participants were first asked about their experiences with barriers to access to DTM before the pandemic (pre-pandemic) and then asked about experiences during the pandemic (mid-pandemic).

**COVID-19 Well-Being.** To assess the impact of the COVID-19 pandemic on participant well-being, we used the 36-item COVID-19 Psychosocial and Practical Experiences (COVID-PPE) scale [33,34]. Participants used a five-point Likert scale (0 = strongly disagree to 4 = strongly agree) to rate the extent to which they agreed with statements in eight domains – anxiety symptoms, depression symptoms, health care disruption, satisfaction with provider response, perceived benefits, social support, perceived stress management, disruption to daily activities and social activities, and financial hardship. Subscale scores were calculated as the mean of all items in each domain (range, 0–4). Higher scores indicated participant experienced ‘more’ of the subscale assessed, e.g., a higher financial hardship subscale score means the participant experienced higher levels of (or worse) financial hardship.

In addition, two composite scores, risk factors and protective factors, were calculated by taking the mean of selected items to assess total amount of negative (risk) and positive (protective) psychosocial outcomes experienced. Risk factors (range, 0–4) was comprised of items from the anxiety symptoms, depression symptoms, health care disruptions, disruption to daily activities & social interactions, and financial hardship subscales. Protective factors (range, 0–4) was comprised of items from the satisfaction with provider response, perceived benefits, social support, and perceived stress management ability subscales. Higher scores for each composite score indicated participants had higher levels of psychosocial outcomes related to COVID-19.

**Demographic Characteristics.** The demographic characteristics of interest selected to characterize the study participants included self-reported community, household and individual level factors identified reported the literature as potentially associated with COVID-19 infection and experiences among Indigenous populations. Individual factors included age (18 to <45, 45 to <65, 65 and older), gender (male or female), education (<high school, high school diploma/GED, ≥ high school diploma/GED), employment (not employed, part or full time), and essential worker status (essential health care, frontline essential, other essential non-essential). Essential workers were defined as those essential to sustain certain businesses which included healthcare operations, essential governmental functions, essential infrastructure activities (e.g., public utilities, school operations), food cultivation, and critical retail (e.g., grocery stores, convenience stores, food banks). Household and community factors included number of persons in the household, number of generations in the household (1, 2, 3 or more generations), housing type (Department of Housing and Urban Development (HUD)/Navajo Housing Authority (NHA), modular house/trailer, traditional), community type (rural, border town, town, urban) and distance to grocery (<15 minutes, 15–30 minutes, 30 minutes to 1 hour,1 hour or more). COVID-19 experiences included testing history for COVID-19 (never tested, ever tested positive, all tests negative), vaccination status (not vaccinated, vaccinated with at least 1 dose) and loss of job or income due to pandemic (yes/no).

### Statistical analysis

Demographic characteristics of study participants were summarized with comparisons between DTM use groups made using t-tests and chi-square tests. Among participants who indicated DTM use, Wilcoxon matched-pairs signed-rank test, a non-parametric test, was used to compare perceived barriers to access DTM before vs. during the COVID pandemic. Barriers to DTM access scores summarized using median and interquartile range (IQR). Participant well-being (COVID-PPE subscales and summary scores) were summarized using means and standard deviations and t-tests used to make comparisons between the two DTM use groups. Analyses were conducted using R statistical software [35].

## Results

The characteristics of Navajo participants in the COVID-19 Impact Survey are shown in Table 1. Most of the survey participants (66.9%) were recruited in-person at flea markets, followed by friends and family (27.4%), newsletters/fliers (11.9%) and social media (10.7%). Age was not reported for 51 participants or one-third (33.3%) of our survey participants, reported age ranged from 18–90 (mean = 44.65, SD = 15.76). Survey participants were mostly female (66.4%) and had at least a high school diploma or GED (84.1%). Most participants lived in modern houses or trailers (46.1% and 31.6%, respectively), with a mean household size of 3.49 persons (SD = 1.78). Participants lived mostly (43.7%) in rural communities and less than 15 minutes driving distance to grocery shopping (30.5%).

### Diné traditional medicine and healing use

Nearly half (n = 74, 48.4%) of participants indicated that they sought DTM from a medicine man, roadman, herbalist, diagnostician, or other traditional practitioner during the pandemic. The majority of DTM users (n = 35, 65.5%) were 18–45 years old while most non-users (n = 27, 56.2%) were 45–65 years older (p < 0.01). Half of non-users (50.6%) reported having post-secondary education compared to over three quarters of DTM users (n = 56, 75.7%). More DTM users (60.0%) reported having part-time or full-time employment than non-DTM users (n = 72, 49.0%) (p < 0.01). Over half of DTM users lived in modern houses (55.4%) compared to just 37.2% among non-DTM users.

### Barriers and access to diné traditional medicine and healing

Table 2 shows the scores for barriers or issues encountered in seeking DTM pre- and mid-pandemic among the 74 participants who reported use of Diné traditional medical care and healing. Overall, median scores remained unchanged from pre-pandemic to mid-pandemic for all barriers or issues except for ‘availability or child or elder care’. The median score for ‘availability of child or elder care’ was higher mid-pandemic than pre-pandemic, but this was not a statistically significant difference.

**Table 2:** Comparison of pre-pandemic and mid-pandemic scores for barriers or issues encountered in Diné traditional medical care and healing.

		PRE pandemic	MID pandemic		
	**N**	**Median (IQR)**	**Median (IQR)**	**Z**	P*
Availability of transportation from home to practitioner.	74	2.00 (1.00-3.75)	2.00 (1.00-3.00)	−1.149	0.251
Availability of money or financial resources.	74	3.00 (1.00-4.00)	3.00 (1.00-3.75)	−1.007	0.314
Availability of traditional medical care or ceremonies at convenient times.	74	3.00 (1.00-4.00)	3.00 (2.00-4.00)	−1.674	0.094
Availability of child or elder care during my care or ceremonies.	73	1.00 (1.00-3.00)	2.00 (1.00-3.00)	−1.951	0.051
I don’t know where to go or who to see.	74	2.00 (1.00-3.00)	2.00 (1.00-3.00)	−0.745	0.456

* based on Wilcoxon matched-pairs signed-rank test.

Of the participants that sought DTM during the pandemic, most indicated that traditional practitioners/healers had implemented measures to address COVID-19 (58.1% with 33.8% strongly agreed and 24.3% agreed). Over half (n = 41) either strongly agreed or agreed (25.7% and 29.7%, respectively) that social distancing guidelines had disrupted or delayed their traditional medicine and healing experiences. Likewise, most participants (25.7% strongly agreed and 30.7% agreed) indicated that Navajo Nation lockdown restrictions had also disrupted or delayed their traditional medical care.

### Diné traditional medicine and healing use and COVID-19 well-being

Comparisons of median scores and standard deviations for all COVID-19 PPE subscales and summary scales between DTM use groups are shown in Table 3. Scores for health care disruption, satisfaction with provider response, disruption to daily and social activities, and financial hardship were uniform between DTM users and non-users. DTM users had slightly higher scores for perceived benefits, social support, and perceived stress management (3.17 (SD = 0.70), 2.89 (SD = 0.96), and 2.76 (SD = 0.80), respectively) but these were not statistically significant. In contrast, significant differences were observed for anxiety and depression scores. DTM users reported higher anxiety symptoms (2.66, SD = 1.06) and depression symptoms (2.47, SD = 1.15) than non-users (2.15 (SD = 1.10) and 1.89 (SD = 1.11), respectively). Both summary scores, risk factors and protective factors, were higher among DTM users than non-users (2.34 vs. 2.00 and 2.90 vs. 2.73, respectively). However, only the risk factors summary score was significantly different (p < 0.05).

**Table 3:** COVID-19 Psychosocial & Practical Experiences (COVID-PPE) Subscales and Summary Scores for Navajo community members by Dine Traditional Medicine and Healing Use.

	Total N = 153	DTM Use 74 (48.7%)	No DTM 79 (51.3%)	
	**Mean (SD)**	**Mean (SD)**	**Mean (SD)**	P*
***Subscales***				
Anxiety Symptoms	2.40 (1.11)	2.66 (1.06)	2.15 (1.10)	<0.01
Depression Symptoms	2.17 (1.16)	2.47 (1.15)	1.89 (1.11)	<0.01
Health Care Disruption	2.03 (1.30)	2.06 (1.34)	1.99 (1.27)	0.75
Satisfaction with Provider Response	2.57 (1.17)	2.58 (1.17)	2.56 (1.17)	0.93
Perceived Benefits	3.10 (0.83)	3.17 (0.70)	3.04 (0.92)	0.33
Social Support	2.78 (0.92)	2.89 (0.96)	2.68 (0.88)	0.17
Perceived Stress Management	2.66 (0.85)	2.76 (0.80)	2.56 (0.90)	0.15
Disruption to Daily Activities & Social Activities	2.02 (1.00)	2.04 (0.93)	2.01 (1.07)	0.83
Financial Hardship	2.01 (1.09)	2.03 (1.04)	2.00 (1.15)	0.87
***Summary Scores***				
COVID-19 Risk Factors Score	2.16 (0.85)	2.34 (0.77)	2.00 (0.90)	0.01
COVID-19 Protective Factors Score	2.81 (0.69)	2.90 (0.65)	2.73 (0.72)	0.13

* based on t-tests

## Discussion

This study was conducted in response to concerns from the Navajo community about the impact of the COVID-19 pandemic including social distancing and lockdown restrictions enacted by the Navajo Nation to reduce transmissions on Diné people’s access, use, and quality of health care including Diné traditional medicine [27]. In this study, we found that nearly half (n = 74, 48.7%) of the study sample reported use of Diné traditional medicine (DTM) or healing during the COVID-19 pandemic. While not directly comparable, a 1998 survey of Navajo Indian Health Service patients found 62% reported ever seeking DTM and 39% reported being regular users [19]. With limited data on current, general population users, it is difficult to interpret our 48.7% during a time of crisis as either an increase of regular users or a decrease of ever users. Nevertheless, DTM users in our sample indicated that the COVID-19 pandemic had minimal impact on the barriers or issues they faced in receiving DTM care or healing and reported higher levels of anxiety, depression, and risk factors than non-users. We cannot know from our analysis whether the observed association between DTM use and mood-related symptoms was because DTM was perceived as a safe and comfortable space during the high anxiety COVID times or if DTM users generally experience more anxiety and depression.

Although several ecological studies have been conducted to describe the factors and vulnerabilities contributing to the increased burden of COVID-19 infection and mortality on the Navajo Nation and other Indigenous communities [12,36–40], these studies were not designed to assess and understand the personal impact of this increased COVID-19 burden on Diné communities. Results from this study contribute to the limited examination of lived experiences and impact of the pandemic from the Indigenous communities’ perspective [41,42].

At the beginning of the pandemic, the Navajo Nation empowered by tribal sovereignty and governance, implemented some of the strictest mitigation strategies in the United States, including mask mandates, restricting social gathering, closing of schools to in-person instruction, weeknight curfews and lockdown restrictions for its citizens [1,26,27]. Social gathering restrictions also extended to in-person traditional ceremonies and other Diné traditional medicine (DTM). Our study findings indicate that these mitigation strategies did not deter Diné people from seeking and receiving Diné traditional medicine and healing. Due to colonization, discrimination, and marginalization, the AI/AN peoples of the US have always faced significant barriers preserving and practicing their culture including the use of traditional Indigenous medicine [18,20,43,44]. Thus, it is likely that the fortitude and adaptability that the Diné people have developed to navigate daily life may have been useful in navigating the pandemic-induced hardships in the pursuit of DTM. For example, members of the Diné Hataałii Association (DHA) adopted technology-based modalities, including phone and online conferencing, in lieu of in-person or home-to-home visits (either within the patient’s or practitioner’s home) for conducting ceremonies and healing practices [25,44,45]. In addition, the DHA shifted member meetings from in-person to online, collected and distributed masks to DHA members across the Navajo Nation, and developed a proclamation informed by Centers for Disease Control and Prevention (CDC) infection control guidelines outlining how to safely proceed in the protection, promotion, and preservation of Diné cultural wisdom and language during the pandemic, despite mitigation restrictions [46]. The Navajo concept of K’é, or family kinship ties, was supportive in the protection of families, communities, elders and cultural keepers facilitating early adoption of masking and social distancing while still taking care of one another.

In contrast to previous studies characterizing users of Indigenous medicine and healing as older and with lower education levels, DTM users in our study were young (56.2% aged 18–45 years old) and had higher levels of education (76.0% reported postsecondary education) [20,43,47]. These findings could be indicative of the resurgence and reclaiming of cultural traditions in younger generations [48–50]. As rebuttal to decades of assimilation and denial of cultural identity, Indigenous communities and tribal nations have identified culture, language, traditional knowledge including traditional medicine as necessary tenets of Indigenous health and well-being [20,48–52]. Although DTM users in our study reported higher levels of anxiety and depression than non-users, they also reported higher levels of protective factors (satisfaction with provider response, perceived benefits, social support, and perceived stress management ability) than non-users. These findings perhaps reflect the emphasis on holistic well-being in Diné culture, wherein shortfalls in mental and emotional health are mitigated through strengthening connections to family, community, nature and non-human kin, and other aspects of life [21–23,53].

This study had several limitations. First, the responses collected are limited by small sample size and potential selection bias. Our sample was slightly older (44.7 y.o. vs 34.6 y.o.), more female (66.4% vs 51%), more educated (84% vs 80% high school diploma or greater) and had higher unemployment (31% vs 15%) compared to the Navajo Nation [54]. While we attempted to reach people throughout the Navajo Nation through the radio and social media most of the respondents were recruited from flea markets in two Navajo communities (Shiprock and Tuba City). These communities are the two most populous on the Navajo Nation, have an economic infrastructure that supports health care and shopping facilities within the community boundaries and have a lower percentage of the population aged 65 and older compared to the overall Navajo Nation (Shiprock: 9%, Tuba City: 12%, Navajo Nation: 14%), thus survey participants may not be representative of the on-reservation Navajo population [54]. Additionally, experiences and perceptions of those that were able to complete surveys (i.e., had internet access or were mobile enough to be recruited at local flea markets) may be different than those that did not participate. Second, our cross-sectional study only captured COVID-19 experiences and perceptions at a single-point in time. Responses were collected between August to October 2021, a period when high vaccination rates and decreased COVID-19 levels on the Navajo Nation had enabled loosening of restrictions on in-person activities and gathering limits. Lastly, our study was primarily focused on examining the influence of proximal factors on individual experiences of the COVID-19 pandemic and may have underestimated the effects of structural vulnerabilities from historical racism and colonialism on these participant’s COVID-19 experiences. Deschine Parkhurst, Huyser and Yellow Horse [40], found ZIP code level COVID-19 incidence among AI/ANs in New Mexico had a stronger association with indices that included measurement of historically embedded vulnerabilities from environmental racism, specifically proximity to abandoned uranium mines compared to current social vulnerability indices that measure the aggregate of specific social, economic, and demographic conditions that place certain populations at risk for hazards (vulnerability) but do not include measures of potential drivers of these vulnerabilities [40].

Future research should explore a diverse range of methods including social media, radio, and community gatherings to recruit and consent Navajo participants from a broad range of backgrounds to examine their perceptions and experiences, during the COVID-19 pandemic. Additionally, future studies could examine assets and resources of Navajo community and participants in contrast to barriers and vulnerabilities to understand how individuals and communities were able to mitigate worsening COVID-19 pandemic conditions.

## Conclusion

In summary, despite significant challenges imposed by the COVID-19 pandemic, this investigation highlighted the incredible resilience of the Diné people and the importance that Diné traditional medicine and healing contributes to overall health and well-being of the Diné people.

## Supporting information

S1 File COVID-19_Community_Questionnaire; Impact of the COVID-19 Pandemic on Navajo Adults and Health Care Providers: Community Questionnaire. (DOCX)

## References

[1] NBC News. How hard-hit Navajo Nation is flattening its coronavirus curve. https://www.nbcnews.com/nightly-news/video/how-hard-hit-navajo-nation-is-flattening-its-coronavirus-curve-89822277664. 2020. Accessed 2022 December 6.

[2] Allen K. Virus strikes at rally: Chilchinbeto church gathering may be source of outbreak. Navajo Times. 2020.

[3] Silverman H, Toropin K, Sidner S, Perrot L. Navajo Nation surpasses New York state for highest Covid-19 infection rate in the US. CNN.com. https://www.cnn.com/2020/05/18/us/navajo-nation-infection-rate-trnd/index.html. 2020. Accessed 2022 August 28.

[4] Navajo Nation Department of Health. COVID-19 Data. https://ndoh.navajo-nsn.gov/COVID-19. 2021. Accessed 2022 August 9.

[5] Hatcher SM, Agnew-Brune C, Anderson M, Zambrano LD, Rose CE, Jim MA, et al. COVID-19 among American Indian and Alaska Native persons — 23 States, January 31–July 3, 2020. MMWR Morb Mortal Wkly Rep. 2020;69(34):1166–9. 32853193 10.15585/mmwr.mm6934e1 PMC7451969

[6] Dong E, Du H, Gardner L. An interactive web-based dashboard to track COVID-19 in real time. Lancet Infect Dis. 2020;20(5):533–4. 32087114 10.1016/S1473-3099(20)30120-1 PMC7159018

[7] Hill M, Houghton F, Hoss MAK. The inequitable impact of Covid-19 among American Indian/Alaskan Native (AI/AN) communities is the direct result of centuries of persecution and racism. J R Soc Med. 2021;114(12):549. 34704844 10.1177/01410768211051710 PMC8722777

[8] Foxworth R, Evans LE, Sanchez GR, Ellenwood C, Roybal CM. I hope to hell nothing goes back to the way it was before: COVID-19, marginalization, and Native Nations. Perspectives on Politics. 2022;20(2):439–56.

[9] Li B, Yang J, Zhao F, Zhi L, Wang X, Liu L, et al. Prevalence and impact of cardiovascular metabolic diseases on COVID-19 in China. Clin Res Cardiol. 2020;109(5):531–8. doi: 10.1007/s00392-020-01626-9 32161990 PMC7087935

[10] Garg S, Kim L, Whitaker M, O’Halloran A, Cummings C, Holstein R, et al. Hospitalization Rates and Characteristics of Patients Hospitalized with Laboratory-Confirmed Coronavirus Disease 2019 - COVID-NET, 14 States, March 1-30, 2020. MMWR Morb Mortal Wkly Rep. 2020;69(15):458–64. doi: 10.15585/mmwr.mm6915e3 32298251 PMC7755063

[11] Cancer Among the Navajo, 2005-2013. Window Rock: Navajo Department of Health NEC. 2014. http://www.nec.navajo-nsn.gov/Portals/0/Reports/CancerAmongNavajo2018Spread.pdf

[12] Kakol M, Upson D, Sood A. Susceptibility of Southwestern American Indian Tribes to Coronavirus Disease 2019 (COVID-19). J Rural Health. 2021;37(1):197–9. doi: 10.1111/jrh.12451 32304251 PMC7264672

[13] Kovich H. Rural matters — coronavirus and the Navajo Nation. N Engl J Med. 2020;383:105–7. 32329970 10.1056/NEJMp2012114

[14] Emerson MA, Montoya T. Confronting Legacies of Structural Racism and Settler Colonialism to Understand COVID-19 Impacts on the Navajo Nation. Am J Public Health. 2021;111(8):1465–9. doi: 10.2105/AJPH.2021.306398 34464207 PMC8489652

[15] Kimani ME, Sarr M, Cuffee Y, Liu C, Webster NS. Associations of race/ethnicity and food insecurity with COVID-19 infection rates across US counties. JAMA Netw Open. 2021;4(6):e2112852. 10.1001/jamanetworkopen.2021.12852 PMC8188266 34100936

[16] Cheng KJG, Sun Y, Monnat SM. COVID-19 Death Rates Are Higher in Rural Counties With Larger Shares of Blacks and Hispanics. The Journal of Rural Health. 2020;36(4):602. 32894612 10.1111/jrh.12511 PMC7885172

[17] US EPA. Navajo Nation: Cleaning Up Abandoned Uranium Mines. https://www.epa.gov/navajo-nation-uranium-cleanup. Accessed 2025 May 27.

[18] Brave Heart MYH, Chase J, Elkins J, Altschul DB. Historical trauma among Indigenous peoples of the Americas: concepts, research, and clinical considerations. J Psychoactive Drugs. 2011;43(4):282–90. 22400458 10.1080/02791072.2011.628913

[19] Kim C, Kwok YS. Navajo use of native healers. Arch Intern Med. 1998;158(20):2245–9. doi: 10.1001/archinte.158.20.2245 9818804

[20] Hillyard V, Ortiz E. As Navajo Nation enforces coronavirus curfew, Arizona sends in the National Guard. NBC News. https://www.nbcnews.com/news/us-news/navajo-nation-enforces-coronavirus-curfew-arizona-sends-national-guard-n1174456. 2020. Accessed 2022 October 14.

[21] Lewis S. Navajo Nation spends weekend on strict 57-hour lockdown as virus death toll rises. CBS News. https://www.cbsnews.com/news/navajo-nation-enters-strict-57-hour-lockdown-coronavirus-pandemic/. 2020. Accessed 2022 October 14.

[22] Redvers N, Blondin B. Traditional Indigenous medicine in North America: A scoping review. PLoS One. 2020;15(8):e0237531. doi: 10.1371/journal.pone.0237531 32790714 PMC7425891

[23] Lewton EL, Bydone V. Identity and healing in three Navajo religious traditions: Sa’ah naagháí bik’eh hózho. Med Anthropol Q. 2000;14(4):476–97. 11224977 10.1525/maq.2000.14.4.476

[24] Schneider GW, DeHaven MJ. Revisiting the Navajo way: lessons for contemporary healing. Perspect Biol Med. 2003;46(3):413–27. 12878811 10.1353/pbm.2003.0044

[25] Begay DH, Maryboy NC. The whole universe is my cathedral: a contemporary Navajo spiritual synthesis. Med Anthropol Q. 2000;14(4):498–520. 11224978 10.1525/maq.2000.14.4.498

[26] King M, Smith A, Gracey M. Indigenous health part 2: the underlying causes of the health gap. Lancet. 2009;374(9683):76–85. doi: 10.1016/S0140-6736(09)60827-8 19577696

[27] Diné Hataałii Association Inc. Diné Hataałii Association, Inc. Available from: https://dhainc.org/

[28] Brown HE, Begay RL, Sanderson PR, Chief C, Lameman B, Harris RB, et al. Healthcare access, attitudes and behaviours among Navajo adults during the COVID-19 pandemic: a cross-sectional study. BMJ Public Health. 2024;2:61. Available from: https://bmjpublichealth.bmj.com 10.1136/bmjph-2023-000061 PMC11636652 39669294

[29] Health Resources & Services Administration. How We Define Rural. https://www.hrsa.gov/rural-health/about-us/what-is-rural. Accessed 2025 May 27.

[30] Saunkeah B, Beans JA, Peercy MT, Hiratsuka VY, Spicer P. Extending Research Protections to Tribal Communities. Am J Bioeth. 2021;21(10):5–12. doi: 10.1080/15265161.2020.1865477 33449863 PMC8280236

[31] Harris PA, Taylor R, Minor BL, Elliott V, Fernandez M, O’Neal L, et al. The REDCap consortium: Building an international community of software platform partners. J Biomed Inform. 2019;95. 10.1016/j.jbi.2019.103208 PMC7254481 31078660

[32] Harris PA, Taylor R, Thielke R, Payne J, Gonzalez N, Conde JG. Research electronic data capture (REDCap)--a metadata-driven methodology and workflow process for providing translational research informatics support. J Biomed Inform. 2009;42(2):377–81. 18929686 10.1016/j.jbi.2008.08.010 PMC2700030

[33] Saez-Clarke E, Otto AK, Prinsloo S, Natori A, Wagner RW, Gomez TI, et al. Development and initial psychometric evaluation of a COVID-related psychosocial experiences questionnaire for cancer survivors. Qual Life Res. 2023;32(12):3475–94. doi: 10.1007/s11136-023-03456-4 37358738 PMC11817160

[34] Penedo F. COVID-19: Impact of the pandemic and HRQoL in cancer patients and survivors (IPHCPS) - Disaster research response (DR2) resources portal. https://tools.niehs.nih.gov/dr2/index.cfm/resource/22131. 2020. Accessed 2022 August 9.

[35] R Core Team. R: A Language and Environment for Statistical Computing. Vienna, Austria: R Foundation for Statistical Computing. 2022.

[36] Yellow Horse AJ, Yang TC, Huyser KR. Structural inequalities established the architecture for COVID-19 pandemic among Native Americans in Arizona: a geographically weighted regression perspective. J Racial Ethn Health Disparities. 2022;9(1):165–75. 33469867 10.1007/s40615-020-00940-2 PMC7815191

[37] Rodriguez-Lonebear D, Barceló NE, Akee R, Carroll SR. American Indian reservations and COVID-19: correlates of early infection rates in the pandemic. Journal of Public Health Management and Practice. 2020;26(4):371–7. 32433389 10.1097/PHH.0000000000001206 PMC7249493

[38] Yellow Horse AJ, Deschine Parkhurst NA, Huyser KR. COVID-19 in New Mexico Tribal Lands: Understanding the Role of Social Vulnerabilities and Historical Racisms. Frontiers in Sociology. 2020;5(610355):1–11. 33869526 10.3389/fsoc.2020.610355 PMC8022442

[39] Denetclaw WF, Otto ZK, Christie S, Allen E, Cruz M, Potter KA. Diné Navajo resilience to the COVID-19 pandemic. PLoS One. 2022;17(8). 10.1371/journal.pone.0272089 PMC9352059 35925907

[40] Deschine Parkhurst NA, Huyser KJ, Yellow Horse AJ. Historical environmental racism, structural inequalities, and Dik’os Ntsaaígíí-19 (COVID-19) on Navajo Nation. J Indig Soc Dev. 2020;9(3):127–40.

[41] Gerald LB, Simmons B, Lowe AA, Liu AH, Nez P, Begay E. COVID-19 on the Navajo Nation: experiences of Diné families of children with asthma. Journal of Asthma. 2023;60(3):565–73. 10.1080/02770903.2022.2073550 PMC13218368 35549973

[42] Kahn CB, James D, George S, Johnson T, Kahn-John M, Teufel-Shone NI, et al. Diné (Navajo) Traditional Knowledge Holders’ Perspective of COVID-19. Int J Environ Res Public Health. 2023;20(4):3728. doi: 10.3390/ijerph20043728 36834423 PMC9964790

[43] Gall A, Leske S, Adams J, Matthews V, Anderson K, Lawler S, et al. Traditional and Complementary Medicine Use Among Indigenous Cancer Patients in Australia, Canada, New Zealand, and the United States: A Systematic Review. Integr Cancer Ther. 2018;17(3):568–81. doi: 10.1177/1534735418775821 29779402 PMC6142081

[44] Chatterjee R. Native Americans come together to protect families from COVID. NPR: Public Health. https://www.npr.org/sections/health-shots/2021/11/24/1058675230/hit-hard-by-covid-native-americans-come-together-to-protect-families-and-elders. 2021. Accessed 2022 December 11.

[45] Wallace AJ. Pandemic highlights coexistence of Navajo traditions and modern times. AZ Mirror. https://azmirror.com/2020/11/12/pandemic-highlights-coexistence-of-navajo-traditions-and-modern-times/. 2020. Accessed 2020 December 11.

[46] Diné Hataałii Association: Traditional Healers and Spiritual Practitioners Leading and Healing Beyond the Pandemic - Indigenous Stories of Strength. https://indigenousstrengths.com/dine-hataalii-association-traditional-healers-and-spiritual-practitioners-leading-and-healing-beyond-the-pandemic. Accessed 2025 May 27.

[47] Gall A, Butler TL, Lawler S, Garvey G. Traditional, complementary and integrative medicine use among Indigenous peoples with diabetes in Australia, Canada, New Zealand and the United States. Aust N Z J Public Health. 2021;45(6):664–71. 34028943 10.1111/1753-6405.13120

[48] Hill DM. Traditional medicine and restoration of wellness strategies. Int J Indig Health. 2009;5(1):26–42.

[49] Barker B, Goodman A, DeBeck K. Reclaiming indigenous identities: culture as strength against suicide among indigenous youth in Canada. Canadian Journal of Public Health. 2017;108(2):e208–10. 10.17269/CJPH.108.5754 PMC6972180 28621659

[50] McCarty TL, Romero ME, Zepeda O. Reclaiming the Gift: Indigenous Youth Counter-Narratives on Native Language Loss and Revitalization. The American Indian Quarterly. 2006;30(1):28–48. doi: 10.1353/aiq.2006.0005

[51] Burnett C, Purkey E, Davison CM, Watson A, Kehoe J, Traviss S, et al. Spirituality, Community Belonging, and Mental Health Outcomes of Indigenous Peoples during the COVID-19 Pandemic. Int J Environ Res Public Health. 2022;19(4):2472. doi: 10.3390/ijerph19042472 35206662 PMC8872600

[52] Watson A, Purkey E, Davison CM, Fu M, Nolan D, Mitchell D. Indigenous strength: Braiding culture, ceremony and community as a response to the COVID-19 pandemic. Int J Indig Health. 2022;17(1).

[53] Storck M, Csordas TJ, Strauss M. Depressive illness and Navajo healing. Med Anthropol Q. 2000;14(4):571–97. 11224981 10.1525/maq.2000.14.4.571

[54] American Community Survey 5-Year Data (2009-2023). https://www.census.gov/data/developers/data-sets/acs-5year.html. Accessed 2025 October 10.

