# Supplementary material for: Diné traditional medicine use and wellbeing among navajo adults during the COVID-19 pandemic: A cross-sectional study
Source: PLoS One. 2025 Dec 5;20(12):e0337427. doi: 10.1371/journal.pone.0337427 (PMC12680193; doi:10.1371/journal.pone.0337427)
Supplement: S1 File — (DOCX) [file pone.0337427.s001.docx]

**COMMUNITY MEMBER Questionnaire**

Welcome and Thank you for considering our study. The project is a collaboration between the University of Arizona and Northern Arizona University. Information from this project will help to understand how the pandemic has influenced access, utilization, and quality of health care for Navajo households and Navajo cancer patients and help the community and health care facilities develop strategies to address these issues.

In this survey, we would like to know the perspectives of individual community members and patients on how the COVID-19 pandemic has influenced care delivery, including cancer prevention, early detection, screening, diagnosis, and treatment, and the interaction of chronic diseases with COVID-19 infection.

Red font indicated formatting and will not be seen by participant.

**INCLUSION CRITERIA**
1. Age ______ years

If <18: Go to Thank you page & participant is excluded from survey,

If >= 18: go to HCW vs Community survey

1. Health Care Workers are defined as anyone who works at a Navajo-centered health care service facility or who works at urban facilities that provide care for Navajo. Does that describe you?

If Yes, go to consent for HCW survey

If No, to consent for this Community survey

1. What is your tribal affiliation? (select all that apply)
2. Navajo
3. Hopi
4. Other
5. None

If None: Go to Thank you page & participant is excluded from survey

If a, b or c, goes to consent for this Community survey

,

1. **COVID-19 EXPERIENCES**

***The following questions ask about your experiences with COVID-19. Please answer the questions to the best of your knowledge; there are no right or wrong answers.***

- 1. Have you been tested for COVID-19? Yes No D/K

If No, skip to Q2

- - - 1. If Yes (tested): How many times were you tested: __ 1-2 times, __3-4 times, __>5 times
      2. If Yes (tested): Were any of the results positive: Yes No D/K
         1. If Yes (positive): When did you first test positive? Month<< year
  1. Did you experience COVID-19 symptoms? Yes No N/A

If No/NA skip to Q3

- - 1. If yes (Symptoms): Did you see a doctor or other provider?
    2. If yes (Symptoms): Were you hospitalized for COVID-19? Yes No N/A
       1. If yes (Hospitalized): How many nights were you in the hospital? __ nights
  1. Did any of the following people in your life experience these COVID-19 related events?

|  | **Tested positive for COVID** | **Hospitalized for COVID** | **Passed Away from COVID** |
| --- | --- | --- | --- |
| 1. Person living in your household | Y / N / DK/ R | Y / N / DK/ R | Y / N / DK/ R |
| 1. Family or relative not living in household | Y / N / DK / R | Y / N / DK / R | Y / N / DK / R |
| 1. Neighbor | Y / N / DK/ R | Y / N / DK/ R | Y / N / DK/ R |
| 1. Friend | Y / N / DK / R | Y / N / DK / R | Y / N / DK / R |
| 1. Co-worker | Y / N / DK/ R | Y / N / DK/ R | Y / N / DK/ R |
| 1. Other community members | Y / N / DK / R | Y / N / DK / R | Y / N / DK / R |

- 1. Have you received the COVID vaccine?
     1. Yes, I have received my first dose.
     2. Yes, I have received my second dose.
     3. No, but I will as soon as I am eligible.
     4. No, and I am not interested.
  2. If a or b or c in (Received Vaccine): Which of the following best describes your reason for getting or wishing to get the COVID vaccine? Choose **only one** option.

1. To reduce my chances of getting the COVID
2. So that I don’t give COVID to other people
3. I am over 65 and/or have a chronic illness
4. The vaccine was easy to get
5. I do not want to miss any work because of the COVID
6. It was recommended to me
7. The vaccine was free for me
8. If d in (Received Vaccine): Which of the following best describes your reason for not wanting to get the COVID vaccine? Choose **only one** option.
9. The vaccine is not effective
10. I do not believe in vaccines
11. I do not like injections
12. The vaccine is not important
13. No time to get the vaccine and/or it was inconvenient
14. Potential side effects
15. I am not afraid of COVID or I don’t think I will get COVID
16. I do not need the vaccine since I do not suffer from a chronic illness or other risk factor
17. The vaccine can cause COVID
18. I already had COVID, so the vaccine is unnecessary
19. **Health Care or Medical Care Related Issues**

***The following questions ask about your health, your experiences with the health care system, and other related issues that may have happened over the past year as you sought care.***

- 1. What is your gender?

1. Male
2. Female
3. Other: _____
   1. Height ____feet __ inches (in)
   2. Weight _______ pounds (lbs)
   3. Have you been told you have any of the following health conditions?

|  | **Yes** | **No** | **Not**  **Sure** |
| --- | --- | --- | --- |
| - - 1. Moderate to severe asthma | 1 | 0 | DK |
| - - 1. Chronic lung disease, such as COPD (chronic obstruction pulmonary disease) | 1 | 0 | DK |
| 1. Obesity (BMI of 30 or higher) | 1 | 0 | DK |
| 1. Cardiovascular disease, such a heart failure or coronary artery disease | 1 | 0 | DK |
| 1. Hypertension or high blood pressure | 1 | 0 | DK |
| 1. Type 2 diabetes | 1 | 0 | DK |
| 1. Type 1 diabetes | 1 | 0 | DK |
| 1. Chronic kidney disease | 1 | 0 | DK |
| 1. Chronic liver disease, including cirrhosis | 1 | 0 | DK |
| 1. Immunocompromised/weakened immune system (including undergoing cancer treatment, organ transplant, prolonged use of corticosteroids and/or HIV) | 1 | 0 | DK |
| 1. Cerebrovascular disease (affects blood vessels and blood supply to the brain) | 1 | 0 | DK |
| 1. Cystic fibrosis | 1 | 0 | DK |
| 1. Neurologic conditions, such as dementia | 1 | 0 | DK |
| 1. Pulmonary fibrosis (having damaged or scarred lung tissue) | 1 | 0 | DK |
| 1. Cancer | 1 | 0 | DK |

1. If Yes (to Cancer): What type: _______
2. If Yes (to Cancer): What stage: _______
3. If Yes (to Cancer): Are you currently in treatment? Y / N
   1. If Yes (to treatment): Which of the following are part of your current treatment plan:

___infusions, ___ surgery ___ radiation

1. Due to COVID-19, did any of the following happen to you:

|  | **Yes** | **No** | **Not**  **Sure** |
| --- | --- | --- | --- |
| 1. Was a scheduled in-person **general medical appointment** cancelled? | 1 | 0 | DK |
| 1. Was a scheduled cancer screening (like a mammogram or endoscopy, or colonoscopy cancelled? | 1 | 0 | DK |
| 1. Did you choose **not** to attend a scheduled in-person **general medical appointment**? | 1 | 0 | DK |
| 1. Did you seek **emergency care** at an urgent care facility or emergency room? | 1 | 0 | DK |
| 1. Did you choose to attend a telehealth appointment for **general medical care.** | 1 | 0 | DK |

- - 1. If Yes, (telehealth): How satisfied are you with your telehealth appointment for **general medical care**?

| **Very Dissatisfied** | **Somewhat Dissatisfied** | **Neutral** | **Somewhat**  **Satisfied** | **Very**  **Satisfied** | **Not Sure** |
| --- | --- | --- | --- | --- | --- |
| 1 | 2 | 3 | 4 | 5 | DK |

1. If Yes (to Cancer): Due to COVID-19 and specifically regarding your cancer care, did any of the following happen to you:

|  | **Yes** | **No** | **Not**  **Sure** |  |
| --- | --- | --- | --- | --- |
| 1. A scheduled in-person **cancer appointment or treatment** was cancelled because of COVID | 1 | 0 | DK |  |
| 1. You chose not to attend a scheduled in-person **cancer appointment or treatment** because of COVID. | 1 | 0 | DK |  |
| 1. You chose to attend a telehealth appointment for **cancer care.** | 1 | 0 | DK | Not An Option |

- 1. If Yes, (to Cancer telehealth): How satisfied are you with your telehealth appointment for **cancer care**?

| **Very Dissatisfied** | **Somewhat Dissatisfied** | **Neutral** | **Somewhat**  **Satisfied** | **Very**  **Satisfied** | **Not Sure** |
| --- | --- | --- | --- | --- | --- |
| 1 | 2 | 3 | 4 | 5 | DK |

1. If Yes (to Cancer): Below is a list of things that other people who receive cancer care (active treatment or follow-up care) said are important**. In the past 7 days, did you feel**

|  | **Not at all** | **A little bit** | **Somewhat** | **Quite a bit** | **Very Much** |
| --- | --- | --- | --- | --- | --- |
| 1. lacking in energy | 0 | 1 | 2 | 3 | 4 |
| 1. pain | 0 | 1 | 2 | 3 | 4 |
| 1. nausea | 0 | 1 | 2 | 3 | 4 |
| 1. worry that your condition will get worse | 0 | 1 | 2 | 3 | 4 |
| 1. rested when you woke | 0 | 1 | 2 | 3 | 4 |
| 1. able to enjoy life | 0 | 1 | 2 | 3 | 4 |
| 1. content with your quality of life right now. | 0 | 1 | 2 | 3 | 4 |

1. **Health Care Disruptions and Concerns (Concerns About Medical Care):**

***The COVID-19 pandemic has impacted many aspects of our daily lives, including access to health care and treatment. The following questions ask about your experiences during the pandemic.***

First, we want to know about **any barriers or issues that you encountered in choosing to receive health care, services or treatment**

1. **BEFORE THE PANDEMIC:** How often did each of the following items influence your decision to receive medical services before the start of the pandemic?

|  | **Never** | **Almost**  **Never** | **Sometimes** | **Almost**  **Always** | **Always** | **Not Sure** |
| --- | --- | --- | --- | --- | --- | --- |
| 1. Availability of transportation from home to medical service. | 1 | 2 | 3 | 4 | 5 | DK |
| 1. Availability of money or financial resources. | 1 | 2 | 3 | 4 | 5 | DK |
| 1. Availability of insurance coverage. | 1 | 2 | 3 | 4 | 5 | DK |
| 1. Availability of medical services or appointments at convenient times. | 1 | 2 | 3 | 4 | 5 | DK |
| 1. Availability of child or elder care during my appointments/services. | 1 | 2 | 3 | 4 | 5 | DK |
| 1. I don’t know where to go to get treated. | 1 | 2 | 3 | 4 | 5 | DK |
| 1. Doctor referral requirements are too much of a hassle or there is a long waiting list. | 1 | 2 | 3 | 4 | 5 | DK |

1. **SINCE THE START OF THE PANDEMIC:** How often did each of the following items influence your decision to receive medical services any time since the pandemic began?

|  | **Never** | **Almost**  **Never** | **Sometimes** | **Almost**  **Always** | **Always** | **Not Sure** |
| --- | --- | --- | --- | --- | --- | --- |
| 1. Availability of transportation from home to medical service. | 1 | 2 | 3 | 4 | 5 | DK |
| 1. Availability of money or financial resources. | 1 | 2 | 3 | 4 | 5 | DK |
| 1. Availability of insurance coverage. | 1 | 2 | 3 | 4 | 5 | DK |
| 1. Availability of medical services or appointments at convenient times. | 1 | 2 | 3 | 4 | 5 | DK |
| 1. Availability of child or elder care during my appointments/services. | 1 | 2 | 3 | 4 | 5 | DK |
| 1. I don’t know where to go to get treated. | 1 | 2 | 3 | 4 | 5 | DK |
| 1. Doctor referral requirements are too much of a hassle or there is a long waiting list. | 1 | 2 | 3 | 4 | 5 | DK |

1. Please indicate the extent to which you agree or disagree with the following statements. **Since the start of the COVID-19 pandemic:**

|  | **Strongly**  **Disagree** | **Disagree** | **Neutral** | **Agree** | **Strongly**  **Agree** | **Not Sure** |
| --- | --- | --- | --- | --- | --- | --- |
| 1. My general medical care has been disrupted or delayed. | 1 | 2 | 3 | 4 | 5 | DK |
| 1. My healthcare providers have taken the necessary measures to address COVID-19. | 1 | 2 | 3 | 4 | 5 | DK |
| 1. If Cancer: My cancer care or follow-up has been disrupted or delayed. | 1 | 2 | 3 | 4 | 5 | DK |
| 1. If Cancer: I received adequate information on prevention, protection, or care for COVID-19 from my cancer care provider. | 1 | 2 | 3 | 4 | 5 | DK |

1. **Diné** **Traditional Medicine and Healing Disruptions and Concerns:**

***The COVID-19 pandemic has impacted many aspects of our daily lives, including access to Diné*** ***traditional medicine and healing, including ceremonies. The following questions ask about your experiences during the pandemic.***

1. Please indicate the extent to which you agree or disagree with the following statements about the impact that social distancing guidelines and Navajo Nation lockdown restrictions had on your ***traditional medicine and healing (such as ceremonies)*** experiences. **Since the start of the COVID-19 pandemic:**

|  | **Strongly**  **Disagree** | **Disagree** | **Neutral** | **Agree** | **Strongly**  **Agree** | **Not Sure** |
| --- | --- | --- | --- | --- | --- | --- |
| 1. Social distancing guidelines (e.g., limiting gathering) have disrupted or delayed me | 1 | 2 | 3 | 4 | 5 | DK |
| 1. Navajo Nation lockdown restrictions have disrupted or delayed me | 1 | 2 | 3 | 4 | 5 | DK |
| 1. My traditional practitioner/healers have taken measures to address COVID-19. | 1 | 2 | 3 | 4 | 5 | DK |
| 1. I have experienced spiritual or psychological hardship/distress due to modifications made to Diné ceremonial interventions because of restrictions and /or lockdowns. | 1 | 2 | 3 | 4 | 5 | DK |

1. During the COVID-19 pandemic, did you seek Diné traditional medical care by any of the following:

|  | **Yes** | **No** | **Not**  **Sure** |
| --- | --- | --- | --- |
| - - 1. Hataali/Medicine Man | 1 | 0 | DK |
| - - 1. Roadman | 1 | 0 | DK |
| 1. Herbalist | 1 | 0 | DK |
| 1. Diagnostician (Hand tremblers or crystal gazers) | 1 | 0 | DK |
| 1. Other Diné ceremonial practitioner | 1 | 0 | DK |

1. If you received Diné traditional medical care by a Diné ceremonial practitioner, Hataali, Roadman, Herbalist, Diagnostician, or other practitioner during the COVID-19 pandemic, please describe below each of the treatments/services/interventions you received:

|  | **Face to face or In Person?** | **Tele or Virtual?**  ***(e.g., phone, Zoom)*** |
| --- | --- | --- |
| 1. Hataali/Medicine Man | Y / N / DK | Y / N / DK |
| 1. Roadman | Y / N / DK | Y / N / DK |
| 1. Herbalist | Y / N / DK | Y / N / DK |
| 1. Diagnostician (Hand tremblers or crystal gazers) | Y / N / DK | Y / N / DK |
| 1. Other Diné ceremonial practitioner | Y / N / DK | Y / N / DK |

Next, we want to know about **any barriers or issues that you encountered in choosing to receive** Diné traditional medical care, services or healing**.**

1. **BEFORE THE PANDEMIC:** How often did each of the following items influence your decision to receive Diné traditional medicine and/or ceremonies before the start of the pandemic?

|  | **Never** | **Almost**  **Never** | **Sometimes** | **Almost**  **Always** | **Always** | **Not Sure** |
| --- | --- | --- | --- | --- | --- | --- |
| 1. Availability of transportation from home to practitioner. | 1 | 2 | 3 | 4 | 5 | DK |
| 1. Availability of money or financial resources. | 1 | 2 | 3 | 4 | 5 | DK |
| 1. Availability of traditional medical care or ceremonies at convenient times. | 1 | 2 | 3 | 4 | 5 | DK |
| 1. Availability of child or elder care during my care or ceremonies. | 1 | 2 | 3 | 4 | 5 | DK |
| 1. I don’t know where to go or who to see. | 1 | 2 | 3 | 4 | 5 | DK |

1. **SINCE THE START OF THE PANDEMIC:** How often did each of the following items influence your decision to receive Diné traditional medicine and/or ceremonies any time since the pandemic began?

|  | **Never** | **Almost**  **Never** | **Sometimes** | **Almost**  **Always** | **Always** | **Not Sure** |
| --- | --- | --- | --- | --- | --- | --- |
| 1. Availability of transportation from home to practitioner. | 1 | 2 | 3 | 4 | 5 | DK |
| 1. Availability of money or financial resources. | 1 | 2 | 3 | 4 | 5 | DK |
| 1. Availability of traditional medical care or ceremonies at convenient times. | 1 | 2 | 3 | 4 | 5 | DK |
| 1. Availability of child or elder care during my care or ceremonies. | 1 | 2 | 3 | 4 | 5 | DK |
| 1. I don’t know where to go or who to see. | 1 | 2 | 3 | 4 | 5 | DK |

1. **COVID-19 SOCIAL AND EMOTIONAL WELLBEING**

***The next questions ask about your thoughts, feelings and support during the last 3 months of the COVID-19 pandemic.*** Please indicate the extent to which you agree or disagree with the following statements.

**In the past 3 months:**

| 1. **COVID-19 Specific Distress (Emotional and Physical Reactions):** | | | | | | |
| --- | --- | --- | --- | --- | --- | --- |
|  | **Strongly**  **Disagree** | **Disagree** | **Neutral** | **Agree** | **Strongly**  **Agree** | **Not Sure** |
| 1. I feel anxious about getting COVID-19 (or if positive: I am anxious about becoming ill). | 1 | 2 | 3 | 4 | 5 | DK |
| 1. I worry about possibly infecting others. | 1 | 2 | 3 | 4 | 5 | DK |
| 1. I am concerned about a family member or close friend getting or dying from COVID-19 |  |  |  |  |  |  |
| 1. I worry about the possibility of dying from COVID-19 | 1 | 2 | 3 | 4 | 5 | DK |
| 1. I feel I have no control over how COVID-19 will impact my life. | 1 | 2 | 3 | 4 | 5 | DK |
| 1. I have experienced feelings of sadness or depression. | 1 | 2 | 3 | 4 | 5 | DK |
| 1. I feel negative and/or anxious about the future. | 1 | 2 | 3 | 4 | 5 | DK |
| 1. I have experienced changes in my sleep. | 1 | 2 | 3 | 4 | 5 | DK |
| 1. I have experienced changes in my eating. | 1 | 2 | 3 | 4 | 5 | DK |
| 1. I have experience difficulty concentrating. | 1 | 2 | 3 | 4 | 5 | DK |
| 1. I have experienced feeling of social isolation or loneliness. | 1 | 2 | 3 | 4 | 5 | DK |
| 1. If Cancer: I fear how the COVID-19 pandemic will impact my cancer care or recovery. | 1 | 2 | 3 | 4 | 5 | DK |
| 1. If Cancer: I am concerned that cancer puts me at a greater risk for being infected or dying from COVID-19. | 1 | 2 | 3 | 4 | 5 | DK |

| 1. **Perceived Benefits:** | | | | | | |
| --- | --- | --- | --- | --- | --- | --- |
|  | **Strongly**  **Disagree** | **Disagree** | **Neutral** | **Agree** | **Strongly**  **Agree** | **Not Sure** |
| 1. I have greater appreciation for my family and close friends. | 1 | 2 | 3 | 4 | 5 | DK |
| 1. I have deeper appreciation for life. | 1 | 2 | 3 | 4 | 5 | DK |
| 1. I have been more grateful for each day. |  |  |  |  |  |  |
| 1. I have been more accepting of things I cannot change. | 1 | 2 | 3 | 4 | 5 | DK |
| 1. I have found new ways of connecting with family and friends. | 1 | 2 | 3 | 4 | 5 | DK |
| 1. If Cancer: I have used my experience in coping with cancer to deal with COVID-19. | 1 | 2 | 3 | 4 | 5 | DK |

| 1. **Functional Support:** | | | | | | |
| --- | --- | --- | --- | --- | --- | --- |
|  | **Strongly**  **Disagree** | **Disagree** | **Neutral** | **Agree** | **Strongly**  **Agree** | **Not Sure** |
| 1. I have received emotional support from family or friends when needed. | 1 | 2 | 3 | 4 | 5 | DK |
| 1. I have received tangible support (e.g., financial, practical) from family or friends when needed. | 1 | 2 | 3 | 4 | 5 | DK |
| 1. I am (or “have been”) there to listen to others’ problems when needed. |  |  |  |  |  |  |
| 1. I have helped others with financial or practical support. | 1 | 2 | 3 | 4 | 5 | DK |

| 1. **Perceived Stress Management (Ability to Manage Stress):** | | | | | | |
| --- | --- | --- | --- | --- | --- | --- |
|  | **Strongly**  **Disagree** | **Disagree** | **Neutral** | **Agree** | **Strongly**  **Agree** | **Not Sure** |
| 1. I am able to recognize thoughts and situations that make me feel stressed or upset about COVID-19. | 1 | 2 | 3 | 4 | 5 | DK |
| 1. I am able to practice relaxation (e.g., deep breathing, meditation) when feeling stress about COVID-19. | 1 | 2 | 3 | 4 | 5 | DK |
| 1. I am able to seek information and plan accordingly to address concerns over the COVID-19 pandemic. |  |  |  |  |  |  |
| 1. I can re-examine negative thoughts and gain a new perspective when concerned about COVID-19. | 1 | 2 | 3 | 4 | 5 | DK |
| 1. I can give myself the caring and tenderness I need. | 1 | 2 | 3 | 4 | 5 | DK |

1. **QUALITY OF LIFE**

***The next questions ask about how the COVID-19 pandemic may have affected your quality of life including your work and the work of others living in your home.*** Please indicate the extent to which you agree or disagree with the following statements. **In the past 3 months:**

| 1. **Disruption to Daily Activities and Social Interactions:** | | | | | | |
| --- | --- | --- | --- | --- | --- | --- |
|  | **Strongly**  **Disagree** | **Disagree** | **Neutral** | **Agree** | **Strongly**  **Agree** | **Not Sure** |
| 1. I have experienced disruptions in day to day social interactions with family and/or friends. | 1 | 2 | 3 | 4 | 5 | DK |
| 1. I have not been able to adequately take care of family members or friends I provide for. | 1 | 2 | 3 | 4 | 5 | DK |
| 1. I have been unable to perform my typical daily routines (e.g., work, physical activity, leisure activity). |  |  |  |  |  |  |
| 1. I have experienced conflict with household members (e.g., spouse/partner, children, parents, others). | 1 | 2 | 3 | 4 | 5 | DK |
| 1. I have had difficulty or been unable to perform my work as usual. | 1 | 2 | 3 | 4 | 5 | DK |
| 1. I have had difficulty taking care of my children’s needs (e.g., providing care, supervising schoolwork) and/or balancing their needs with other responsibilities. | 1 | 2 | 3 | 4 | 5 | DK |

| 1. **Financial Hardship:** | | | | | | |
| --- | --- | --- | --- | --- | --- | --- |
|  | **Strongly**  **Disagree** | **Disagree** | **Neutral** | **Agree** | **Strongly**  **Agree** | **Not Sure** |
| 1. I have experienced financial difficulties. | 1 | 2 | 3 | 4 | 5 | DK |
| 1. I have not been able to purchase or obtain basic necessities (e.g., food, personal care products). | 1 | 2 | 3 | 4 | 5 | DK |
| 1. I have been anxious about losing or having lost my job, or my primary source of income. | 1 | 2 | 3 | 4 | 5 | DK |
| 1. I have not been able to adequately provide for others I financially support. | 1 | 2 | 3 | 4 | 5 | DK |
| 1. I feel anxious about being able to maintain or not having adequate health care insurance. | 1 | 2 | 3 | 4 | 5 | DK |

1. Did you lose your job or primary source of income due to COVID-19? Yes No N/A
2. Did any member of your household lose their job or primary source of income? Yes No N/A
3. If currently employed, are you: (select all that apply)

___ working at home ___ physically going to work ­­­­­­­____ going and staying at work for extended shifts

___ N/A __ other (please explain)

1. Does your job put you in direct physical contact with other people?

__ Frequently ___ Sometimes ­­­­­­­___ Never N/A

1. Due to COVID-19, our household income has:

__ Decreased __ Increased __ Not changed

1. If your income decreased, what was the reason (check as many as apply):

__ Loss of work

__ Worked fewer hours because of the employer decision

__ Worked fewer hours because I needed to take care of family

__ I needed to share finances with other family/ friends

__ Inability to work at home

__ Other

1. If your income increased, what was the reason (check as many as apply):

__ Started a new job __ Family member started new job __ My work became busier __ Other

1. What type of employed work do you do? **Check all that apply**
   1. Agriculture/farming
   2. Grocery
   3. Restaurant/food service
   4. Retail
   5. Warehouse or processing
   6. Public safety
   7. Education
   8. Transportation
   9. Health care or other medical services
   10. Utility worker
   11. Hospitality
   12. Construction
   13. Other
2. Does your employer provide or implement any COVID-19 safety precautions (such as mask, sanitizer, protective shields, limiting number of people, etc)? Yes No N/A
3. When working, how safe do you feel with these precautions? [insert Likert scale]
4. Do any members of your household work in a job that puts them in direct contact with other people? Yes No N/A
5. How often are you spending time outside your home?

__ No time __ once a week __ every 2-3 days __ normal routine

1. In general, do you feel that you are accomplishing more or less (e.g., activities, tasks, hobbies, interests)? ___More ___ Less ___ Same
2. If there is anything you’d further like to let us know about how COVID had impacted your life, please do so here:
3. **Household and Community Information**

***This section is information about you, your household and your community. This helps us know we are getting a broad range of your community’s experience.***

- 1. Which of the following best describes your housing structure:
     1. Modular house / trailer
     2. Modern (HUD housing)
     3. Traditional (Hogan)
     4. Traditional (Rock)
     5. Other: _____
  2. Which of the following best describes the fuel source used most commonly in your house structure for heating?
     1. Coal only
     2. Coal and wood
     3. Wood only
     4. Electric
     5. Gas
     6. Propane
     7. Other: _____
  3. How many people INCLUDING yourself are currently living in your home? ____
  4. How many individuals in each relationship group live with you?

Me and: __ grandparents

__ parents

__ kids

__ grandchildren

__ siblings (brother or sister)

__ uncles/aunts

__ spouse/partner

__ other non-related persons

- 1. What is the highest level of school you have completed?
     1. Grade 1-8
     2. Grades 9-11/Some high school
     3. Grade 12/HS Diploma or GED
     4. Some college, Technical degree or Associates degree
     5. Bachelor’s degree
     6. Master’s or higher
     7. Other
  2. Where do you currently live? City/Chapter/Village ____, State ____
  3. How would you describe your community?

1. Urban (in a large city)
2. Town (in a smaller city or town)
3. Border Town
4. Rural (not many homes nearby, small town)

- 1. About how long is a one-way trip for groceries from your home?
     1. Less than 15 minutes
     2. 15 to 30 minutes
     3. 30 minutes to 1 hour
     4. 1 hour or more
  2. During the pandemic, for groceries (check all that apply)
     1. My family has been bringing me groceries
     2. I am receiving food boxes
     3. I am still getting my own groceries at the store
     4. I am doing curb-side pickup from my grocery store
     5. I am having groceries delivered (e.g., Instacart, Amazon, Grocery store delivery)
  3. Which of these sources best describes your primary drinking water source?
     1. Well water or water from community spigot
     2. NTUA tap water
     3. NTUA tap with additional in-home filter (e.g., Brita filter or pitcher)
     4. Bottled/delivered water
  4. Do you have internet in your home?
     1. Yes, I have access to stable reliable internet
     2. Yes, but unstable
     3. Yes, but only when restricting number of users
     4. No I do not.

Thank you for participating in this study. If you have questions, please reach out to the investigators. To provide information so we can send you your compensation for completing this questionnaire, please click here: Add Link for Compensation. Please note that the information you provide for the compensation will not be linked to the answers you’ve provided.

In the future, our team may seek to link COVID-19 experiences with longer term cancer or chronic disease outcomes. If you are willing to be contacted and for this survey information to be linked to that future project, please click here: Add Link for Secure REDCap database linking Name, Email address/ Phone and Study ID. Please note that the information you provide for future work will be stored in a separate secure file. It will include only the Study Reference Number identifying your responses not the responses themselves as well as the Contact information you provide.

We are also conducting semi-structured interviews/talking circles with a few members of the community who would like to share their COVID-19 experiences. We expect that telephone interview to last no more than 2 hours. Your participation in this survey is in no way impacted by your choice to participate in the semi-structured interview/talking circle. If you are interested in more information, please click here: Add Recruitment Link for Focus Group. Please note that the information you provide for the information will not be linked to the answers you’ve provided in this questionnaire.
